# Intestinal-Type Adenocarcinoma of the Urinary Bladder With Coexisting Cystitis Cystica et Glandularis and Intestinal Metaplasia: A Histopathological Case Report

**DOI:** 10.7759/cureus.36554

**Published:** 2023-03-22

**Authors:** Hristo Popov, George S Stoyanov, Peter Ghenev

**Affiliations:** 1 General and Clinical Pathology, Forensic Medicine and Deontology, Medical University of Varna, Varna, BGR; 2 Pathology, Complex Oncology Center, Shumen, BGR

**Keywords:** urology, histopathology, cystitis cystica et glandularis, adenocarcinoma, urinary bladder

## Abstract

Adenocarcinomas of the urinary bladder are exceedingly rare and present in various morphological forms. Virtually all of these are identical to glandular malignant neoplasia native to topographically neighboring organs, where the incidence of adenocarcinoma is also much more common, such as the large intestine. Cases of glandular malignancies of the urinary bladder, therefore, require not only a detailed histopathological evaluation and interpretation but also a detailed clinical and radiological one. These should be performed with the goal of proving the origin of the tumor as one arising from the urinary bladder and not an entry originating from another organ and invading or producing metastasis to it. A controversial etiopathogenic link to urinary bladder adenocarcinoma is that of* cystitis cystica et glandularis*, which often coexists with the condition. Herein, we present a case report of non-muscle-invasive urinary bladder adenocarcinoma in a previously healthy male patient in his forties with a known history of *cystitis cystica et glandularis*. The patient presented with gross hematuria, and based on his known urological condition, a cystoscopy with biopsy was performed, showing submucosal proliferation of atypical glands. The detailed clinical and radiological evaluation showed no evidence of malignancy at other sites. As the malignancy was non-muscle-invasive, an intravesical dose of the Bacillus Calmette-Guérin vaccine was administered. The patient was followed up with cystoscopy, and a biopsy showed no evidence of residual malignancy, with *cystitis cystica et glandularis* persisting. A year following the diagnosis, the patient is still actively monitored, and no recurrence is noted.

## Introduction

Urothelial carcinomas are the predominant malignant tumor developing in the urinary bladder worldwide [1]. Other malignancies, such as squamous cell carcinoma, develop significantly less often and with a geographical predominance, while primary bladder adenocarcinoma is exceedingly rare. This, together with the proximity of the bladder to organs in which adenocarcinoma is not a native and common entity, such as the colon, often creates diagnostic difficulties as to the exact origin of the tumor [2]. 

Urinary bladder adenocarcinoma shows a varied histological pattern and, rather than a distinct entry, represents a histogenic group of malignancies with overlapping histological and immunohistochemical profile for multiple types of adenocarcinoma as well as varying differentiation, sometimes masking the glandular characteristics of these tumors. 

Therefore, not only a histopathological but also a detailed clinical and radiological evaluation is needed for the diagnosis and exclusion of invasive and metastatic adenocarcinomas to the urinary bladder from other primary organ sites as both the prognosis and patient treatment vary significantly [3]. Clinical, imaging, histologic, and immunohistochemical correlations should be made in making this diagnosis, as the prognosis and therapeutic options for primary versus metastatic adenocarcinoma vary greatly [4].

The most common variant of urinary bladder adenocarcinoma is the intestinal (columnar) type, while other types such as mucinous, clear cell, hepatoid, signet ring, mixed, and not otherwise specified variants are also included in the current classification [1,5].

*Cystitis cystica et glandularis*, superficial acinar, and intestinal metaplasia in the adjacent urothelium are usually encountered together with adenocarcinoma, although they are not classically regarded as risk factors for the development of this histogenic group of malignancies.

This histogenic group of malignancies usually develops in patients over 60 with presenting symptoms of hematuria; the gross morphology is usually that of a single, relatively small lesion, predominantly located in the trigone, although the dome of the bladder is also a common location [6,7].

## Case presentation

**Figure 1:**
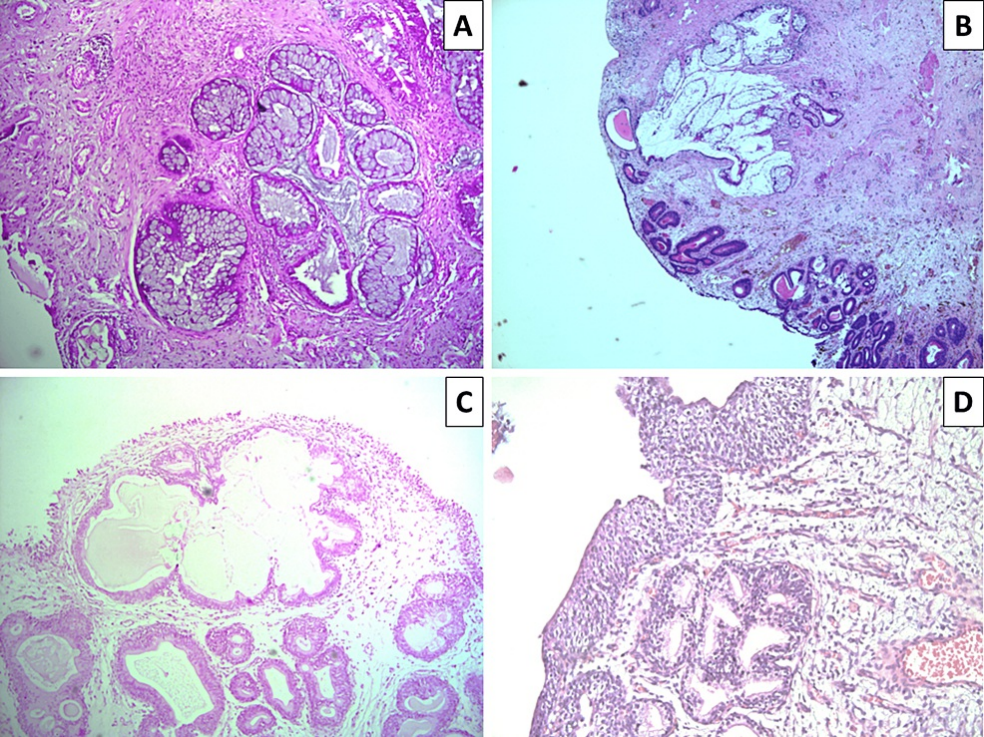
Cystitis cystica et glandularis. Submucosal glandular metaplasia and cyst formation, hematoxylin and eosin stain, original magnifications x50 (A and B) and x100 (C and D).

A previously healthy male in his forties presented to our institution with complaints of painless gross hematuria for the past several weeks. The laboratory tests returned within reference values, and the ultrasound revealed no abnormalities. Cystoscopy was negative for significant findings, and multiple pinch biopsies were obtained from the bladder mucus. Histopathology revealed a classical constellation of *cystitis cystica et glandularis* (Figure 1).

**Figure 2:**
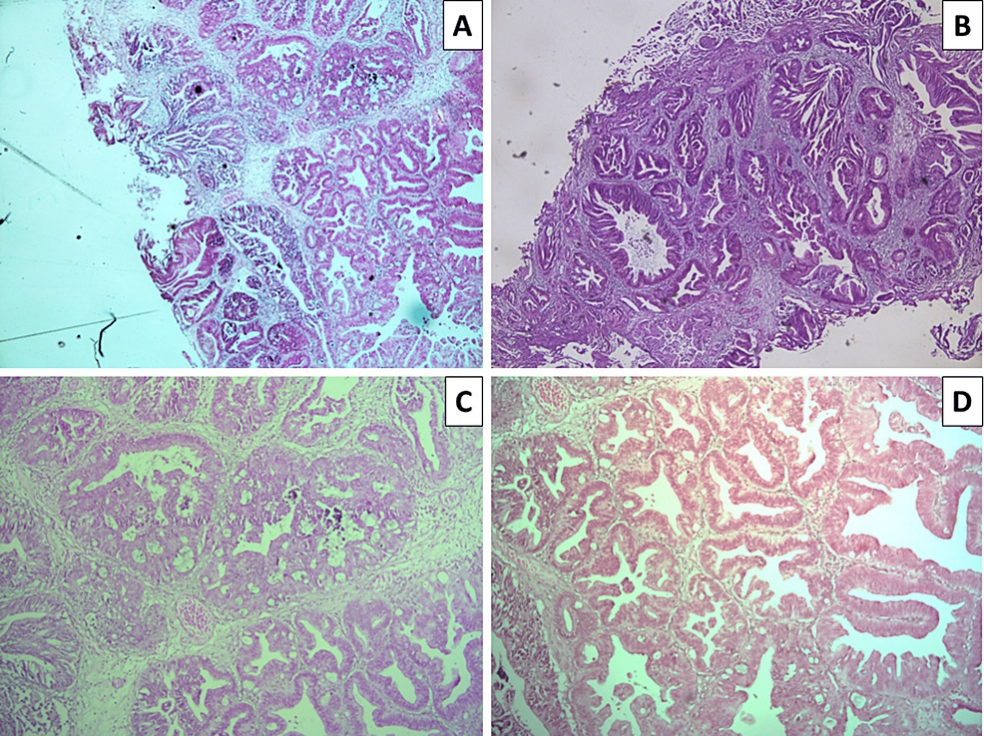
Intestinal-type adenocarcinoma of the urinary bladder. Atypical glandular structures within the submucosa, hematoxylin and eosin stain, original magnifications x20 (A) and x50 (B-D).

The patient was referred to an outpatient urologist, followed up with annual cystoscopies, and remained stable for the next four calendar years. Upon presentation for a scheduled cystoscopy, the patient reported steadily increasing gross hematuria over the past month. Cystoscopy showed an elevated and granular patch of mucosa in the trigone, and multiple pinch biopsies were obtained. Histopathology again showed changes associated with *cystitis cystica et glandularis* and submucosal proliferation of atypical glandular structures composed of atypical columnar intestinal-type malignant cells, a moderately differentiated adenocarcinoma (Figure 2).

The patient was referred for further clinical and radiological evaluation, including intestinal endoscopy, abdominal ultrasound, and chest and abdominal computer axial tomography, with the goal of excluding the presence of a primary gastrointestinal tract malignancy. The tumor was accepted as primary intestinal-type adenocarcinoma of the urinary bladder. As the tumor was present only in the submucosa, the patient was administered an intravesical dose of Bacillus Calmette-Guérin (BCG) and referred for cystoscopy two months later. Target biopsies were obtained from the same places on the repeat cystoscopy, and no evidence of adenocarcinoma was noted, while *cystitis cystica et glandularis* with intestinal metaplasia persisted.

One year later, the patient is monitored every six months with cystoscopy and biopsy, and no recurrence has been noted.

## Discussion

According to the fifth edition of the World Health Organization (WHO) classification of urinary and male genital tumors, adenocarcinoma of the urinary bladder, classified as not otherwise specified (NOS), accounts for around 0.5-2% of all malignant bladder tumors [5]. Other rare types of glandular malignancies, such as urachal-derived adenocarcinoma and diverticular adenocarcinoma, account for less than one case per 1,000,000 capita annually [1]. Despite the histomorphological overlap between these entities, our case was considered as adenocarcinoma of the urinary bladder, NOS, as urachal adenocarcinomas require a urachal remnant to develop and are typically located in the urinary bladder dome, while diverticular adenocarcinomas develop in diverticula [1]. As no evidence for such was present in the presented patient and, furthermore, the tumor was located within the trigone of the bladder, we excluded these entries as possible. While a subset of conventional urothelial malignancies does present with focal glandular differentiation, no such differentiation was noted in our case, with the tumor not having a connection to the superficial urothelial mucosa and being wholly located in the submucosa [1,5].

Regarding the immunophenotype of these tumors, they closely resemble their gastrointestinal counterparts. Both groups are typically cytokeratin (CK) 20, caudal type homeobox transcription factor 2 (CDX2) and vilin positive, although some cases of bladder adenocarcinoma can be CDX2 and vilin negative [1,5]. Guanine-adenine-thymine-adenine-binding protein 3 (GATA3), although a good marker for urothelial differentiation and origin, is often times negative, while CK7 shows variable positivity, with β-catenin also being variably positive, although less than the reported frequency for colorectal adenocarcinoma [5]. Uroplakin, while highly specific for urothelial differentiation, is also a marker that can be both negative in glandular malignancies of the bladder and positive in colorectal ones as well [1].

Hence, the differential diagnosis between primary bladder adenocarcinoma and adenocarcinoma metastasis/invasion/infiltration to the urinary bladder is difficult and, depending on the case, impossible based on immunohistochemistry alone, and these distinct groups require significant clinical and radiological workup to differentiate between them [3,8,9].

Due to the lack of native glandular epithelium within the urinary bladder, there are several branching and overlapping theories on the development of glandular malignancies within it, focusing on different aspects of cell biology. Some authors suggest mechanisms based on urothelial dedifferentiation and the development of glandular properties, although these tumors at least preserve some urothelial morphologies [1]. Despite the co-occurrence of cystitis cystica et glandularis and adenocarcinomas of the bladder, the etiopathogenic connection between the two is poorly established. Some authors believe that bladder adenocarcinoma arises due to the development of intestinal metaplasia on the background of chronic irritation and inflammation, while others suggest that it arises from remnants of endodermal intestinal tissue. While both theories are relative to the development of malignant tumors in general, the latter is well supported by the presence of urachal remnants and the development of urachal-type adenocarcinomas, which develop in the urinary bladder and tend to develop in the dome area and not the trigone as seen in our case [10]. Other reported risk factors include *Schistosoma haematobium* infection, villous adenoma, and cystocele [9,11-14].

Treatment options, especially in non-muscle-invasive forms, are controversial. As seen in our case, an intravesical dose of BCG vaccine proved efficient, although many patients may not respond to it, and other options such as chemotherapy and radiotherapy may prove more efficient [3,8,15].

In our case, despite immunohistochemistry for the aforementioned markers not being performed, the patient underwent extensive clinical and radiological evaluation, which did not detect a tumor outside the bladder. Despite its low reported incidence of occurring in around 2-4% of all colorectal malignancies, based on their high incidence rate overall, it becomes evident that invasion and metastasis significantly outweigh primary bladder adenocarcinomas in their occurrence [1,5,16,17].

Our case responded well to conservative treatment based on the submucosal location of the tumor and followed the conventional monitoring for patients with urothelial malignancies, while many cases present at significantly more advanced stages and the consensus for treatment is cystectomy [1,5]. 

## Conclusions

The patient presented was diagnosed with *cystitis cystica et glandularis* and, four years later, with intestinal-type adenocarcinoma of the urinary bladder. These malignancies are a challenge not only to the pathologist but also in a clinical context, as there needs to be a clinical differentiation between adenocarcinoma invasion and metastasis, predominantly from the gastrointestinal tract. Unlike in many other instances, the immunohistochemical evaluation does not aid in the differentiation of the primary site. Therefore, these tumors necessitate the exclusion of secondary bladder adenocarcinomas by clinical, imaging, endoscopic, and biopsy means.
